# Adolescents' mutual acculturation attitudes and their association with national self-identification in three Swiss cantons

**DOI:** 10.3389/fsoc.2023.953914

**Published:** 2023-06-21

**Authors:** Petra Sidler

**Affiliations:** ^1^Institute for Research and Development, School of Education, University of Applied Sciences and Arts Northwestern Switzerland, Windisch, Switzerland; ^2^National Centre of Competence in Research (NCCR)–On the move, University of Neuchâtel, Neuchâtel, Switzerland; ^3^Culture, Society, and Behavior Lab, Department of Psychology, University of Oslo, Oslo, Norway

**Keywords:** mutual acculturation, majority acculturation, acculturation attitudes, Switzerland, adolescence, latent profile analysis (LPA), national self-identification

## Abstract

Acculturation is a mutual process, meaning that members of minority as well as majority groups acculturate and thus experience cultural and psychological changes when having intercultural contact. This study assessed mutual acculturation attitudes in the school context through a four-dimensional measurement examining attitudes toward (1) migration background students' heritage culture maintenance and their (2) dominant culture adoption, (3) majority students' intercultural knowledge acquisition, and (4) schools' intercultural contact endorsement. Acculturation attitudes are commonly analyzed through minority and majority perspectives; however, the ways in which researchers categorize group members can differ significantly from how those members self-identify. This matters particularly for adolescents because they explore group identities and belongings. So far, adolescents' *mutual acculturation attitudes* have not been studied in relation to national self-identification measures. The current study addressed this research gap by analyzing mutual acculturation attitudes in relation to how strongly adolescents self-identify as (1) being Swiss, (2) having a migration background, and (3) the interaction of the two. The sample consisted of 319 adolescents in public secondary schools in three German-speaking cantons in Switzerland (45% female, *M*_age_ = 13.60 years, range 12–16). Latent profile analyses resulted in three distinct mutual acculturation profiles. The first is a *mutual integration profile* (*n* = 147, 46%), where minority and majority adolescents and schools are expected to integrate. The second is a *multiculturalism* profile (*n* = 137, 43%), with slightly lower expectations in all dimensions. The third is a *cultural distancing* profile (*n* = 33, 10%), which places particularly low expectations on majority adolescents and schools. Through an analysis of variance and a multiple logistic regression, those in the *cultural distancing* profile were found to identify significantly stronger as not having a migration background compared to those in the *mutual integration* profile. Thus, students having separation expectations toward minority students and non-involvement expectations toward schools and majority students are more likely to self-identify as not having a migration background than students having mutual integration expectations.

## 1. Introduction

Global migratory flows have resulted in societies becoming more culturally diverse. In 2020, almost 281,000,000 individuals resided outside of their countries of birth, accounting for 3.6 percent of the global population [International Organization for Migration (IOM) United Nations., 2022]. Nonetheless, migrants constitute a fraction of the worldwide population; as of 2020, the remaining 96.4 percent of the world's population resided in the nations where they were born. Migratory flows frequently result in intercultural contact between individuals and groups with varied languages, lifestyles, faiths, and customs. Such intercultural contact takes place wherever people meet. For adolescents, this is mainly at school, in their neighborhood, among their peers, and within their families. The cultural and psychological changes that people and communities undergo because of intercultural contact is defined as *acculturation* (Berry, 2019). According to an ecological approach (Bronfenbrenner, 1979), institutions such as schools interact with and are rooted in national settings. As a result, global migratory patterns not only make society more culturally varied, but also its institutions, such as schools. Thus, nation-states and schools are both crucial contexts for adolescents' acculturation (Motti-Stefanidi et al., 2012) and their national as well as ethnic identity development (Kotowski, 2013). The latter matters particularly for adolescents because they explore group identities and feelings of belonging in various contexts (Phinney, 1992; Portes and Rivas, 2011).

Acculturation is the subject of extensive research, with the initial term dating back to 1936 and referring to first-hand intercultural encounter that results in behavioral and attitudinal changes (Redfield et al., 1936). Anthropologists and sociologists were among the first to investigate acculturation, particularly focusing on the dynamics of distinct cultural groups coming into ongoing contact (Burgess and Park, 1933; Redfield et al., 1936; Boas, 1948). Several decades later, social psychologists began to investigate the issue. Psychologists, on the other hand, were more concerned in the individual level, and therefore focused on how people, rather than groups, respond to changes in their cultural context (Rudmin, 2009). The common focus of this extensive research on acculturation is minority group members' acculturation. Thus, the main aim has been concerned with how ethnic minorities or immigrants adjust to their host country (e.g., Berry et al., 1989), or how majority group members expect minority group members to acculturate (e.g., Bourhis et al., 1997). Only very recently did researchers start to study majority group members' acculturation (Lefringhausen, 2015; Haugen and Kunst, 2017; Kunst et al., 2021). This new line of research looks at how incorporating parts of minority cultures affects the dominant culture. However, investigating either minority or majority acculturation (i.e., how either minority group members or majority group members acculturate) provides only one side of the narrative. To recognize the interactive character and hence mutuality of the acculturation process (Berry, 2009; Chirkov, 2009), it is key to assess the acculturation of minority and majority group members concurrently. Thus, *mutual acculturation* refers to how both minority and majority group members acculturate. Moreover, following an ecological perspective (Bronfenbrenner, 1979), not only do individuals adjust to a change in cultural context but also do institutions. Institutions should be adjusted to take the needs of all individuals and cultural groups living together into account (Berry, 2019). This is particularly important in schools, as their diversity policies do not only set the foundation for broader narratives for inclusion and exclusion in society, but diversity policies also play a key part in accommodating students with and without migration backgrounds (Celeste et al., 2019). Schools are therefore conceptualized as being an acculturation context while also being an acculturating agent that chooses whether and how it adjusts to heterogeneity in school classes. Thus, in this study mutual acculturation attitudes were assessed on a four-dimensional scale measuring attitudes toward (1) heritage culture maintenance of migration-background adolescents, (2) dominant culture adoption of migration-background adolescents, (3) intercultural knowledge acquisition of majority adolescents, and (4) intercultural contact endorsement of schools (for measurement validation see Sidler et al., 2021).

Acculturation attitudes are commonly analyzed through minority and majority perspectives and group categorizations are essential to make diverse experiences and attitudes evident (Criado-Perez, 2019). However, study results may vary depending on the type of categorization chosen. Therefore, it is key to use self-identification, country of birth, nationality, as well as migration and generation status with caution (Moffitt and Juang, 2019). This means that the ways in which researchers differentiate between minority and majority group members can be challenging. The concept *migration background* is often used to this end, which often relies on nationality and countries of birth of an individual and its parents (Horvath, 2019). When assessing differences between those with and without migration backgrounds, researchers face the methodological challenge of grouping participants and defining what it means to have a migration background. However, tackling this challenge through variables like nationality and place of birth may lead to categorizing generations of families as having a migration background, implying that they can never fully arrive in their country of residence (El-Tayeb, 2014). Moreover, categorizing also means fixing something that is fluid; migration movements vary over time and thus identities and feelings of belongings may also vary. Nevertheless, assessing how experiences and opportunities differ according to migration background may be key in discovering the root of discrimination; what escapes categorization probably also escapes detection (Criado-Perez, 2019). Unfortunately, using such terms might also lead to stigmatizing, stereotyping, and impinging negatively on study participants (Moffitt et al., 2020). Moreover, how researchers categorize group members can differ significantly from how the group's members self-identify (Horvath, 2019). Self-identification measures evaluate self-perception and self-categorization based on experienced intercultural relations, which may differ from ascribed categorizations. Therefore, the key is to study not only the acculturation attitudes of young minority and majority members through concepts such as migration background, nationality, place of birth, ethnic groups, and migrant generation but also through self-identification. So far, *mutual acculturation attitudes* have not been studied in relation to self-identification. This study addresses this research gap by analyzing mutual acculturation attitudes in relation to adolescents' national self-identification. The latter was assessed by adolescents' self-identification as (1) being Swiss, as (2) having a migration background, and (3) the interaction of the two.

To clarify, the terms *minority* and *majority* are used in this study to reflect social power hierarchies (Connell, 1998). Thus, the term majority relates to the dominant group and the term minority relates to non-dominant groups in a society and therefore not necessarily to a numerical majority or minority. Such a differentiation is important as it reflects the political climate on immigrant integration and immigrant rights in Switzerland. For example, foreign nationals are excluded from active and passive voting rights on the federal level as well as on most cantonal and communal levels in Switzerland, and thus are not allowed to actively shape the political decisions affecting their lives (Blatter et al., 2017). Thus, Swiss nationals are considered a dominant majority in the sense that they have a right to shape the political decisions for themselves as well as for foreign nationals. Moreover, following integration policies in the Federal Act on Foreign Nationals, SR 142.20 (2005), integration is understood as an effort solely put on foreign nationals. Thus, again, Swiss nationals are a dominant majority to which foreign nationals must adapt to. Thus, in this study, *minority acculturation* relates to the acculturation of individuals with a migration or ethnic minority background (not because they are fewer in numbers, but because they have less power in the political society), and *majority acculturation* relates to the acculturation of individuals possessing the Swiss nationality as well as Swiss institutions.

## 2. Mutual acculturation at school

Following the tradition of cross-cultural psychology, acculturation is a process of ongoing cultural and psychological change resulting from direct or remote intercultural contact (Ferguson and Bornstein, 2012; Berry, 2019). Acculturation entails the Latin words *ad cultura*, which mean “leading to a culture” (Zick, 2010). Culture is a fluid and dynamic construct of a groups' shared meanings, understandings, and referents (Shore, 2002), along with specific artifacts and behaviors (Rudmin, 2009). The acculturation framework includes acculturation conditions, attitudes, and outcomes (Arends-Tóth and Van de Vijver, 2006). Acculturation conditions refer to individual and contextual characteristics, whereas acculturation attitudes relate to preferences on how individuals or groups acculturate. Acculturation conditions and attitudes lead to outcomes such as psychological, emotional, and sociocultural adjustment. Acculturation attitudes have been assessed for minority acculturation (e.g., Berry et al., 1989; Bourhis et al., 1997), majority acculturation (e.g., Haugen and Kunst, 2017; Kunst et al., 2021), and mutual acculturation (Sidler et al., 2021, 2022). This study assessed mutual acculturation attitudes of adolescents in the school context and focused therefore on acculturation conditions and acculturation attitudes of Arends-Tóth and Van de Vijver (2006) theoretical model.

Attitudes toward minority acculturation are commonly assessed using a bidimensional model. Berry et al. (1989) combined minority group members' heritage culture maintenance and having relationships with other groups, which led to four acculturation strategies. Bourhis et al. (1997) developed the model further into combining minority group members' heritage culture maintenance and their dominant culture adoption. Bourhis et al. (1997) proposed to assess both dimensions from majority as well as minority group members' perspectives. Through the combination of two dimensions that concern minority group members' heritage culture maintenance and dominant or mainstream culture adoption, four acculturation strategies or expectations emerged: *integration/multiculturalism* (agreement with both dimensions), *assimilation/melting pot* (agreement with adoption, disagreement with maintenance), *separation/segregation* (agreement with maintenance, disagreement with adoption), and *marginalization/exclusion* (disagreement with both dimensions; Bourhis et al., 1997).

As these two dimensions concerning minority acculturation can be assessed from minority and majority group members' perspectives (Bourhis et al., 1997), acculturation orientations of minority group members that concern their own acculturation and majority group members' acculturation expectations of minority group members can be measured. Attitudes toward majority acculturation have been assessed concerning whether aspects of minority cultures were incorporated in the dominant culture and, if so, how they were incorporated (Haugen and Kunst, 2017). A recent review found strategies concerning majority group members' acculturation orientations that included integration, separation, assimilation, marginalization, and diffusion (Kunst et al., 2021). Yet, assessing either minority or majority acculturation examines only one side of the story. Mutual acculturation, in the sense that all groups are interacting and adjusting when facing intercultural contact, relates to assessing the two perspectives at stake toward all groups acculturating due to experiencing a change in cultural context. To illustrate, expecting minority group members to integrate while majority group members are not expected to integrate is a different finding than if both groups are expected to integrate in the wider society. Thus, mutual acculturation relates to assessing the common effort between minority and majority group members concerning mutual social and cultural inclusion.

The school setting is recognized as a key environment for adolescents' continuous acculturation (Horenczyk and Tatar, 2012). Adolescents' mutual acculturation attitudes at school have been assessed through a four-dimensional model that combines minority and majority group members' perspectives on minority and majority acculturation in the school context (Sidler et al., 2021). Intercultural interactions and communications require the acquisition of new intercultural skills by both minority and majority students (Landis and Bhawuk, 2020), implying that both minority and majority adolescents adjust to the intercultural context at school and thus acculturate. As acculturation means “leading to a culture” (Zick, 2010), intercultural knowledge acquisition is a first acculturating step. Intercultural knowledge refers first to the cognitive awareness that there are distinctive cultural orientations, contexts, and characteristics and, second, to knowing how such cultural contexts, characteristics, and orientations are being distinguished. Notably, to adopt a specific cultural characteristic, one must acquire some sort of knowledge about it first. Also, to develop intercultural competence, i.e., to develop the cognitive, affective, and behavioral skills and characteristics necessary to have appropriate and effective intercultural interactions (Bennett, 2013), the acquisition of intercultural knowledge is a first necessary step. Thus, majority students' acculturation is conceptualized as intercultural knowledge acquisition. Next to majority students, schools are also considered to be an agent within the realm of majority acculturation, because they are a national institution with prevalent power ideologies (Warikoo and Carter, 2009). Berry (2019) has pointed out that national institutions like schools must be adjusted to better meet the needs of all groups and individuals now living together. Thus, next to being an acculturation context, schools are also cultural actors. Schools have agency in the sense that they shape the environment for intercultural contact and learning through school diversity policies implementation (Celeste et al., 2019; Schwarzenthal et al., 2020). Schools may raise awareness concerning stereotypes and implicit bias (Warikoo et al., 2016), or they may choose to ignore the facticity of intercultural contact and acculturation at school. Specifically, whether and how schools handle cultural diversity has an impact on both minority and majority adolescents' educational outcomes and belonging (Baysu et al., 2021) as well as their intercultural competence development (Schwarzenthal et al., 2020). The way in which schools frame or refrain from framing cultural diversity matters because adolescent students develop intergroup attitudes and intercultural skills (Raabe and Beelmann, 2011). Therefore, schools' diversity policies set the basis for broader narratives concerning intercultural relations. Thus, in shaping cultural diversity policies and creating diversity climates, schools make a choice between taking an active or a passive part in the acculturation process.

## 3. Acculturation and categorization

To assess how attitudes toward minority, majority, or mutual acculturation differ between minority and majority group members, researchers face a categorization challenge. Reflecting on the link between the use of categorizations and the authority of interpretation is key because researchers, as well as policy makers, play a part in continuing social norms concerning national belonging (Moffitt and Juang, 2019). Following a constructivist perspective, categorizations implicitly constitute the groups they describe (Brubaker, 2009). This means that categorizations such as migration backgrounds and ethnic and racial labels are socially constructed and not a naturally given fact in the real world. However, not only do perceived and ascribed belongings to socially constructed groups have a real impact on people (Elias and Feagin, 2020) but also categories such as nationalities; the Quality of Nationality Index (2018) shows how nationality impacts individuals' personal and professional trajectories. However, terminology matters because categorizations risk misrepresenting immigrants and their descendants by depicting them as a uniform group compared to an essentialized, non-immigrant national group (Moffitt and Juang, 2019). Group labels may lead to discursive but biased boundaries, which ultimately affect policy strategies and the daily lives of potentially miscategorized individuals. Moreover, categorizations may relate to exclusionary notions of national identity and impose societal and historical power structures on individuals (Elrick and Farah Schwartzman, 2015). Self-identification presents an alternative group categorization. Self-identification measures shift the agency from the receiving society to individuals with or without supposed migration backgrounds, allowing them to define themselves according to how they identify in that moment. Self-identification measures thus account for the fluidity of not only identities but also concepts such as migration background that are officially treated as being more objective. Using national terms inclusively and including all individuals who self-identify as a part of a nation helps shift the construction of national belonging to better reflect the contemporary population (Moffitt and Juang, 2019). In the German context, 80% of individuals who have heritage outside of Germany identify as German (Foroutan et al., 2014); however, when the national label *German* is used, many of them are excluded (Moffitt and Juang, 2019). Self-identification measures allow individuals to decide for themselves how they are described, regardless of how much their self-perceptions rely on societal power structures. Preferred group labels may change over time and across social contexts, particularly for adolescents, who develop their own labels and try out various identities (Portes and Rivas, 2011). Self-identification can be assessed in relation to ethnic and national groups as well as in relation to being a migrant, refugee, or having a migration background. This study focuses on national self-identification on two continuous scales, so that study participants can describe themselves both as being Swiss and as having a migration background simultaneously. Assessing the combination of the national self-identification measures means acknowledging that simultaneous self-identification as being Swiss and having a migration background is not a contradiction and therefore that national self-identifications are nuanced.

## 4. Acculturation context

According to the eco-developmental model of human development, adolescents' development is embedded in their surrounding environment (Bronfenbrenner, 1979). Their surroundings consist of a variety of microsystems such as the family, peer group, and school, which are important settings not only for adolescents' development but also their acculturation. Furthermore, in accordance with (Bronfenbrenner, 1979) ecological approach, microsystems are embedded in the larger social environment, implying that schools are incorporated in national contexts while interacting with them. Acculturation experiences are influenced by the migration, integration, and diversity policies and atmospheres of both national governments and schools, making them both essential settings for adolescents' acculturation (Motti-Stefanidi et al., 2012). In acculturation research, the contextual approach stresses that the link between acculturation and acculturation outcomes like adjustment is defined by the surrounding contexts (Birman and Simon, 2014) and their interactions (Makarova et al., 2021).

Switzerland is an interesting case study; It is a federal system consisting of 26 cantons of which 6 are half cantons. Moreover, it has four official languages and therefore four linguistic regions, with various cantons being bilingual. The school system in Switzerland consists of 1–2 years of kindergarten (age four to six), followed by 6 years of primary school (age 6–12), and 3 years of secondary school (age 12–15) with each canton having different names for the diverse school levels (Educationsuisse, n.d.). The specific curriculums used to be defined by each canton independently. In 2014, however, the so-called Lehrplan21 was introduced for German-speaking and bilingual cantons: It defines a shared curriculum that should be implemented until 2021 [Deutschschweizer Erziehungsdirektoren-Konferenz (D-EDK), 2014]. The Lehrplan21 is legally effective in all three cantons assessed in this study.

Concerning cultural diversity, Switzerland had about 2,550,00 (30% of the total population) foreign-born residents in 2019 (Organisation for Economic Co-operation Development, 2021). A culturally diverse population means that schools and teachers are challenged to integrate students with diverse cultural backgrounds (Makarova, 2019). The Migrant Integration Policy Index (Migrant Integration Policy Index, 2020) examines integration policies of various countries and creates a multidimensional picture of migrants' opportunities to participate in a given society. Following the 2019 assessment of the Migrant Integration Policy Index (2020), Switzerland's integration approach was classified as “temporary integration,” meaning that while foreign residents can receive targeted assistance to get equal opportunities, they lack the long-term security concerning permanent stay and to participate as full citizens. Overall, Switzerland scored 50 on the MIPEX 100-point scale and was assessed to have slightly unfavorable policies concerning anti-discrimination and access to nationality.

Then, concerning education, the MIPEX assessed how accessible education is for students with a migration background, whether there is teacher training concerning dealing with cultural diversity at school, and whether special needs of migrant students are being considered. Switzerland scored 48 on the 100-point scale and the MIPEX stressed that Swiss schools must address issues of segregation and diversity at school. Moreover, as Switzerland was found to have high naturalization barriers (Migrant Integration Policy Index, 2020), many foreign residents may self-identify as Swiss because of how long they have resided in the country.

Taking a closer look at federal law tackling integration in Switzerland, it becomes evident that whereas integration policies in Switzerland expect openness from the Swiss majority, the policies' regulatory focus lies on the integration of foreign nationals (Federal Act on Foreign Nationals, 2005). Such a conceptualization bases on the assumption that Swiss nationals are already inherently part of the Swiss society, whereas foreign nationals must be integrated into it. However, the Swiss society foreign nationals should integrate into may be mainly imagined (Anderson, 2006). Following the OECD (2023), Switzerland is a multicultural society. Therefore, it seems likely that not only foreign nationals, but also Swiss nationals are challenged to integrate into the modern multicultural Swiss society. However, Swiss law putting this responsibility mainly on foreign nationals reflects power dynamics between the Swiss majority and diverse non-Swiss minorities.

## 5. The present study

How adolescents' mutual acculturation attitudes differ in relation to their national self-identification presented a research gap. Thus, this study's main aim was to assess whether adolescents in Swiss schools differ in their mutual acculturation attitudes based on their self-identification as being Swiss, as having a migration background, and the interaction of the two. Three research questions guided the analysis:

(1) Do national self-identification measures differ from other categorizations like students' nationality and place of birth as well as parents' places of birth?
(2) How many and what kind of mutual acculturation profiles are found through latent profile analyses?
(3) Do these mutual acculturation profiles differ according to students' national self-identification as being Swiss, as having a migration background, and the interaction of the two?

The analytic strategy consisted of three steps: First, it was of interest to assess whether categorizations based on national self-identification differ from other categorizations like nationality and places of birth. Therefore, self-identification as being Swiss and as having a migration background were compared to students' nationality and place of birth as well as their parents' place of birth through frequencies, crosstabs, and correlations. Then, through latent profile analyses, I inductively identified mutual acculturation profiles. Finally, through an analysis of variance (ANOVA) and multiple logistic regressions, I examined these mutual acculturation profiles in relation to adolescents' self-identification as (1) being Swiss, (2) having a migration background, and (3) their interaction.

## 6. Methods

### 6.1. Participants

The study participants were eighth graders from 30 classes in 19 schools in the middle and lower secondary levels in three German-speaking cantons of Switzerland—namely, Aargau, Basel-Stadt, and Solothurn. In total, 319 students (45% female, 54% male, 1% other, *M*_age_ = 13.60 years, *SD* = 0.67, age range 12–16 years) from rural and urban regions participated in the data collection. Given the presence of first-generation immigrant students and following intercultural consulting, the questionnaires were translated into five additional languages (Arabic, English, Farsi, French, and Turkish) through a content translation using a culturally sensitive approach and the four-eyes principle (Peña, 2007). Of the students, 95% filled in the questionnaire in German.

### 6.2. Procedure

The data used for this study were collected in 2020 during the second data collection within the longitudinal project Overcoming Inequalities with Education: School and Resilience of the national center of competencies in research, NCCR–On the Move, in Switzerland. During the COVID-19 pandemic, Switzerland has closed its schools only once during spring 2020 (Leybold-Johnson, 2021), allowing for data collection in schools in autumn. Research assistants collected the data through a web-based questionnaire at schools during class hours. They instructed students, answered questions, and wrote a protocol for each data collection session. It took students ~1 h to fill in the questionnaires on tablets.

The sampling started in 2019, after ethical approval by the Ethics Committee of the University of Zurich. After having contacted cantonal educational offices, the research team contacted school principals and class teachers via telephone and email. Teachers informed parents and students about the study and asked both for written consent. The self-identification measures were introduced in the data collection in 2020, which is why the data from 2020 are used in this study.

The sample size was not defined by an a priori power analysis and, therefore, it is a convenient random sample. As many schools as possible were contacted to recruit full classes. However, the gender and immigrant compositions of the sample (45% female, *n* = 143; 57% Swiss, *n* = 182) were comparable with official statistics of students within the relevant school tracks and cantons [Federal Statistical Office (FSO), 2020a,b].

### 6.3. Measures

Mutual acculturation attitudes were assessed using four dimensions, each comprising seven items (see Annex Table A1 in the annex for all items verbatim). The measurement has been validated with data from the first data collection in 2019 (Sidler et al., 2021). I measured the students' attitudes on a 4-point Likert scale ranging from 1 (*disagree*), 2 (*somewhat disagree*), 3 (*somewhat agree*), to 4 (*agree*). Thus, higher scores relate to higher agreement with the relevant dimension. The four dimensions consist of two for minority acculturation and two for majority acculturation. Thus, I measured attitudes toward (a) *migration-background students' heritage culture maintenance* (e.g., “I find that it is important for teenagers from another country who live in Switzerland to be allowed to preserve their languages”; Cronbach's α = 0.87 and McDonald's ω = 0.86); (b) *migration background students' dominant culture adoption* (e.g., “I find that it is important for teenagers from other countries who live in Switzerland to adopt the dominant traditions and customs of Switzerland”; Cronbach's α and McDonald's ω both = 0.89); (c) *majority students' acquisition of cultural knowledge* (e.g., “I find that it is important for Swiss teenagers who live in Switzerland to get to know the ways of life of teenagers from other countries who live in Switzerland”; Cronbach's α and McDonald's ω both = 0.92); and (d) *schools' endorsement of intercultural contact* (e.g., “I find that it is important for the Swiss schooling system to allow teenagers from other countries and Swiss teenagers to exchange information about religions”; Cronbach's α and McDonald's ω both = 0.94). Internal consistency measures were very high across the four dimensions, meaning that the seven items in each dimension yielded similar scores. Given the multi-level structure of the data, intraclass correlations were assessed on two (students—school classes; students—schools) and three levels (students—school classes—schools) in MPlus 8.3 (Muthén and Muthén, 1998–2019). Intraclass correlations were all lower than 0.100, meaning that there was only small variance at both the school and class level.

Self-identification was assessed through two dimensions, each consisting of one item that was rated on a 4-point Likert scale ranging from 1 (*yes*), 2 (*somewhat yes*), and 3 (*somewhat no*) to 4 (*no*). The first dimension assessed *Swiss self-identification* (“Would you consider yourself to be Swiss?”) and the second dimension assessed *migration background self-identification* (“Would you consider yourself to have a migration background?”). The first item has been reverse-coded, meaning that the higher the mean in each of the two dimensions, the more students consider themselves to be Swiss (4; *yes*) or the less they consider themselves to have a migration background (4; *no*).

Students' nationality was assessed through two questions: First, assessing what nationality students possess with 1 (*Swiss*) and 0 (*other, specify*). Second, whether students possess any further nationalities, with 1 (*no*) and 0 (*yes, specify*).

Students' place of birth was assessed through one question asking where they have been born with 1 (*Switzerland*) and 2 (*other, specify*).

Parents' place of birth was assessed through one question asking where their parents have been born with 1 (*both parents have been born in Switzerland*), 2 (*one parent has been born in Switzerland and one parent has been born abroad*), 3 (*both parents have been born abroad*), and 4 (*I don't know*).

Study participants reported their gender as either “girl,” “boy,” or “other.” Following theories on dominant masculinities (Connell, 1998), a dummy variable (female or other = 0, male = 1) was used for data analysis.

### 6.4. Data analysis

This study had two goals: First, to examine whether national self-identification measures differed in relation to measures of nationality and places of birth of students and their parents. To this end, frequencies, crosstabs, and correlations were run in SPSS (Version 27). Then, the main aim of this study was to assess whether adolescents' mutual acculturation attitudes differed according to their self-identification as being Swiss as well as having a migration background. In a first step, latent profile analysis (LPA) was used to identify mutual acculturation profiles in MPlus 8.3 (Muthén and Muthén, 1998–2019). LPA is a person-centric and typological statistical analysis, like latent class analysis, but it uses continuous instead of categorical indicators. LPA reveals latent groups from observed data (Oberski, 2016) that would not have been discovered through variable-centric analyses (Ferguson et al., 2020). The underlying assumption of LPA is that “people can be *typed* with varying degrees of probabilities into categories (subpopulations) that have different configural profiles of personal and/or environmental attributes” (Spurk et al., 2020, p. 1–2). This means that hidden typologies are formed based on the probability that each participant belongs to a specific pattern. Thus, study respondents are empirically typed into categories based on observations that appear to be similar (Hagenaars and McCutcheon, 2002). These profiles are defined through low variability within a profile as well as high variability among profiles (Masyn, 2013). Moreover, LPA was found to be a parsimonious method of modeling acculturation without expecting profiles in advance (Fox et al., 2013). Thus, the main advantage of using LPA to assess mutual acculturation profiles lies in exploring the data and therefore in fitting mutual acculturation profiles to the data instead of fitting the data into pre-expected profiles.

Specifically, LPA with continuous indicators of mutual acculturation consisting of minority as well as majority acculturation were conducted to identify the best-fitting solution. Models were analyzed with up to seven latent profiles and maximum likelihood estimation with robust standard errors (MLR) was applied. Missing data was handled using full information maximum likelihood (FIML), except for when values were missing on all variables (*n* = 2, 1%). Following Dong and Peng (2013), FIML is preferable to other methods because it does not ascribe missing data and therefore uses only available data. All models were estimated with 2,000 random start values and 500 iterations, and the 100 best solutions were retained. Means and variances were freely estimated in all profiles and models (Morin et al., 2016). Model fit indices and theoretical considerations concerning the meaningfulness of the profiles and their theoretical interpretability guided model selection (Geiser, 2009). Concerning model fit values like the Akaike information criterion (AIC), the Bayesian information criterion (BIC), the sample-size adjusted BIC (aBIC), and the log likelihood, a lower number indicated a better fit. An elbow graph helps assessing gradient changes concerning model fit improvements when comparing a K-profile model with a K-1 profile model. Entropy reflects the precision of the classification (with 0 for a low accuracy and 1 for a high accuracy) and values over 0.7 are regarded as adequate (Lanza and Cooper, 2016). Then, a significant Lo-Mendell-Rubin Adjusted Likelihood Ratio test (aLMR-LRT; Lo et al., 2001) and Bootstrapped likelihood Ratio test (BLRT; McLachlan et al., 2019) indicate the best fitting solution when comparing a K-profile model with a K-1 profile model.

To assess differences according to self-identification as being Swiss or as having a migration background, LPAs were run for four subsamples based on self-identification as being Swiss (those who identify as somewhat Swiss and those who don't) or as having a migration background (those who identify as somewhat having a migration background and those who don't). However, concerning the LPA results based on self-identification as being Swiss as well as having a migration background, the two-profile-solutions did not have a significant better model fit than the one-profile-solution for none of the four subsamples (Swiss, non-Swiss, migration background, and non-migration background; see Annex Tables A2, A3). The Lo–Mendell–Rubin adjusted log-likelihood-ratio test (aLMR) was not significant for any of the seven profile solutions for the non-Swiss and the migration background self-identifying subsamples, possibly due to low sample sizes. For the Swiss and the non-migration background self-identifying subsamples, only the three-profile-solution showed a significant aLMR test.

Thus, to assess differences in mutual acculturation attitudes in relation to self-identification, LPAs were assessed for the full sample in a first step. Then, in a second step, univariate ANOVAs were run in SPSS (Version 27) to assess group differences based on self-identification concerning (1) being Swiss, and (2) having a migration background. ANOVAs test whether a specific variable's mean value differs among various independent groups. To confirm the ANOVA and to take the interaction between the two self-identification variables into account, multiple logistic regressions were used to investigate whether self-identification as being Swiss might moderate the effects of self-identification as not having a migration background on the found mutual acculturation profiles. Both predictors were centered around their means (Aiken et al., 1991) before computing the interaction term, and all variables were entered into the model together.

## 7. Results

### 7.1. Descriptive statistics

Descriptive statistics were assessed to answer the first research question. Table 1 displays frequencies and a crosstab of the two self-identification measures. Table 2 shows means, standard deviations, and correlations of the four acculturation dimensions, the two self-identification measures, students' nationality, and country of birth of students and their parents. Initial checks for any gender differences concerning the four mutual acculturation dimensions and the two self-identification measures were conducted but showed to be not significant.

**Table 1:** Self-identification frequencies and crosstab: being swiss vs. having a migration background (*n* = 297).

		**Self-identification having a migration background**			
		**Yes, 20% (*****n*** = **64)**	**Somewhat yes, 12% (*****n*** = **38)**	**Somewhat no, 17% (*****n*** = **53)**	**No, 45% (*****n*** = **142)**
Self-identification being Swiss	Yes 42% (*n* = 135)	5% (*n* = 14)	2% (*n* = 5)	6% (*n* = 18)	31% (*n* = 91)
	Somewhat yes 25% (*n* = 78)	4% (*n* = 11)	5% (*n* = 14)	8% (*n* = 23)	9% (*n* = 28)
	Somewhat no 19% (*n* = 59)	8% (*n* = 24)	4% (*n* = 12)	3% (*n* = 8)	3% (*n* = 10)
	No, 14% (*n* = 44)	5% (*n* = 15)	2% (*n* = 7)	1% (*n* = 4)	4% (*n* = 13)

In total, there were 1% (n = 3) missing answers in Swiss self-identification and 7% (n = 22) missing answers in migration background self-identification.

**Table 2:** Means, standard deviations, and correlations of mutual acculturation attitudes, self-identification, nationality, and country of birth.

	***n***	***M***	***SD***	**Migration- background students' heritage culture maintenance**	**Migration- background students' dominant culture adoption**	**Majority students' acquisition of cultural knowledge**	**Schools' endorsement of intercultural contact**	**Self-identification as being Swiss**	**Self-identification as not having a migration background**	**Having Swiss Nationality**	**Student born in SWI**	**Both Parents born in SWI**	**One Parent born in SWI**
Migration background students' heritage culture maintenance	308	3.42	0.63	1 (*n* = 308)									
Migration background students' dominant culture adoption	288	2.38	0.82	−0.001 (*n* = 285)	1 (*n* = 288)								
Majority students' acquisition of cultural knowledge	300	2.93	0.79	0.375^***^ (*n* = 293)	0.215^***^ (*n* = 276)	1 (*n* = 300)							
Schools' endorsement of intercultural contact	298	3.22	0.75	0.466^***^ (*n* = 294)	0.071 (*n* = 277)	0.651^***^ (*n* = 287)	1 (*n* = 298)						
Self-identification as being Swiss	316	2.96	1.08	−0.128^*^ (*n* = 307)	0.118^*^ (*n* = 287)	−0.154^**^ (*n* = 298)	−0.051 (*n* = 297)	1 (*n* = 316)					
Self-identification as not having a migration background	297	2.92	1.21	−0.109 (*n* = 290)	−0.014 (*n* = 276)	−0.207^***^ (*n* = 282)	−0.149^*^ (*n* = 282)	0.406^***^ (*n* = 297)	1 (*n* = 297)				
Having Swiss Nationality	319	na	na	−0.110 (*n* = 308)	0.019 (*n* = 288)	−0.108 (*n* = 300)	−0.041 (*n* = 298)	0.627^***^ (*n* = 316)	0.328^***^ (*n* = 297)	1 (*n* = 319)			
Student born in SWI	319	na	na	0.016 (*n* = 308)	0.043 (*n* = 288)	−0.080 (*n* = 300)	−0.057 (*n* = 298)	0.361^***^ (*n* = 316)	0.133^*^ (*n* = 297)	0.422^***^ (*n* = 319)	1 (*n* = 319)		
Both Parents born in SWI	319	na	na	−0.135^*^ (*n* = 308)	0.049 (*n* = 288)	−0.178^**^ (*n* = 300)	−0.158^**^ (*n* = 298)	0.621^***^ (*n* = 316)	0.458^***^ (*n* = 297)	0.619^***^ (*n* = 319)	0.346^***^ (*n* = 319)	1 (*n* = 319)	
One Parent born in SWI	319	na	na	0.042 (*n* = 308)	0.019 (*n* = 288)	0.111 (*n* = 300)	0.135^*^ (*n* = 298)	−0.150^**^ (*n* = 316)	−0.107 (*n* = 297)	−0.043 (*n* = 319)	0.010 (*n* = 319)	−0.382^***^ (*n* = 319)	1 (*n* = 319)
Both Parents born outside SWI	319	na	na	0.138^*^ (*n* = 308)	−0.076 (*n* = 288)	0.102 (*n* = 300)	0.046 (*n* = 298)	−0.497^***^ (*n* = 316)	−0.376^***^ (*n* = 297)	−0.562^***^ (*n* = 319)	−0.313^***^ (*n* = 319)	−0.653^***^ (*n* = 319)	−0.387^***^ (*n* = 319)

^*^p ≤ 0.05. ^**^p ≤ 0.01. ^***^p ≤ 0.001. SWI, Switzerland; na, not applicable. The four acculturation dimensions and the self-identification measures are assessed on a 4-point Likert scale from disagree/no (1) to agree/yes (4). Thus, concerning the acculturation dimensions, higher means indicate stronger agreement with the respective dimension. Concerning self-identification measures, higher means indicate stronger identification as Swiss or as not having a migration background. Students' nationality and country of birth as well as country of birth of parents have been dummy-coded as (0) not applicable and (1) applicable.

Self-identification means were highly similar among the two single self-identification measures. Yet, looking at the self-identification crosstab, many small groups emerged, showing that the two categories “Swiss” and “migration background” are not mutually exclusive (see Table 1). Nearly one third self-identified as being Swiss while not having a migration background. Yet, only 5% self-identified as not being Swiss while having a migration background. Thus, national self-identification exists in diverse combinations.

Crosstabs between the two self-identification measures and students' nationality as well as students and their parents' country of birth were analyzed (see Annex Figures A1–A6). Overall, 42% of the participants did not possess Swiss nationality^1^ and 19% have been born outside Switzerland. Both parents of 40% of the participants had been born outside Switzerland, and 19% of the participants had only one parent who had been born outside Switzerland. Most students who possessed the Swiss nationality, who have been born in Switzerland, or whose parents have both been born in Switzerland self-identified as (somewhat) being Swiss as well as (somewhat) not having a migration background. However, the graphs also show diversity in terms of self-identification and nationality as well as country of birth. For example, many students who have been born in Switzerland also self-identified as (somewhat) not being Swiss or as (somewhat) having a migration background. On the other hand, some students who have been born outside of Switzerland self-identified as (somewhat) being Swiss or as (somewhat) not having a migration background. There were students with Swiss nationality who self-identified as (somewhat) having a migration background or as (somewhat) not being Swiss, while there were students without Swiss nationality who self-identified as (somewhat) not having migration background or as (somewhat) being Swiss.

Descriptive statistics were also assessed for the four mutual acculturation dimensions. Data showed the strongest agreement for the first and the fourth dimensions—namely, migration-background students' heritage culture maintenance and schools' endorsement of intercultural contact. Then, data showed a strong tendency to agree concerning the third dimension, majority students' acquisition of cultural knowledge. However, data showed a slight tendency to disagree concerning the second dimension, migration-background students' dominant culture adoption.

Table 2 also displays correlations among the four acculturation dimensions, the two self-identification measures and students' nationality as well as students' and parents' countries of birth. No multicollinearity was detected. The strongest significant positive correlations emerged between the two majority acculturation dimensions and between students having Swiss nationality, both parents having been born in Switzerland, and students self-identifying as being Swiss. One of the strongest significant negative correlations emerged between both parents being born outside Switzerland and students having Swiss nationality. A relatively strong positive correlation emerged between self-identifying as Swiss and self-identifying as not having a migration background, reflecting the 31% self-identifying as being Swiss while not having a migration background. Weak and moderate predominantly negative correlations emerged between the self-identification measures and the four acculturation dimensions: The more students self-identified as either Swiss or as not having a migration background, the less they agreed with migration-background students' heritage culture maintenance, majority students' acquisition of cultural knowledge, and schools' endorsement of intercultural contact; however, these students tended to agree with migration-background students' dominant culture adoption. However, opposite to the two self-identification measures, students' nationality and place of birth did not correlate significantly with the four acculturation dimensions.

### 7.2. Mutual acculturation profiles

**Figure 1:**
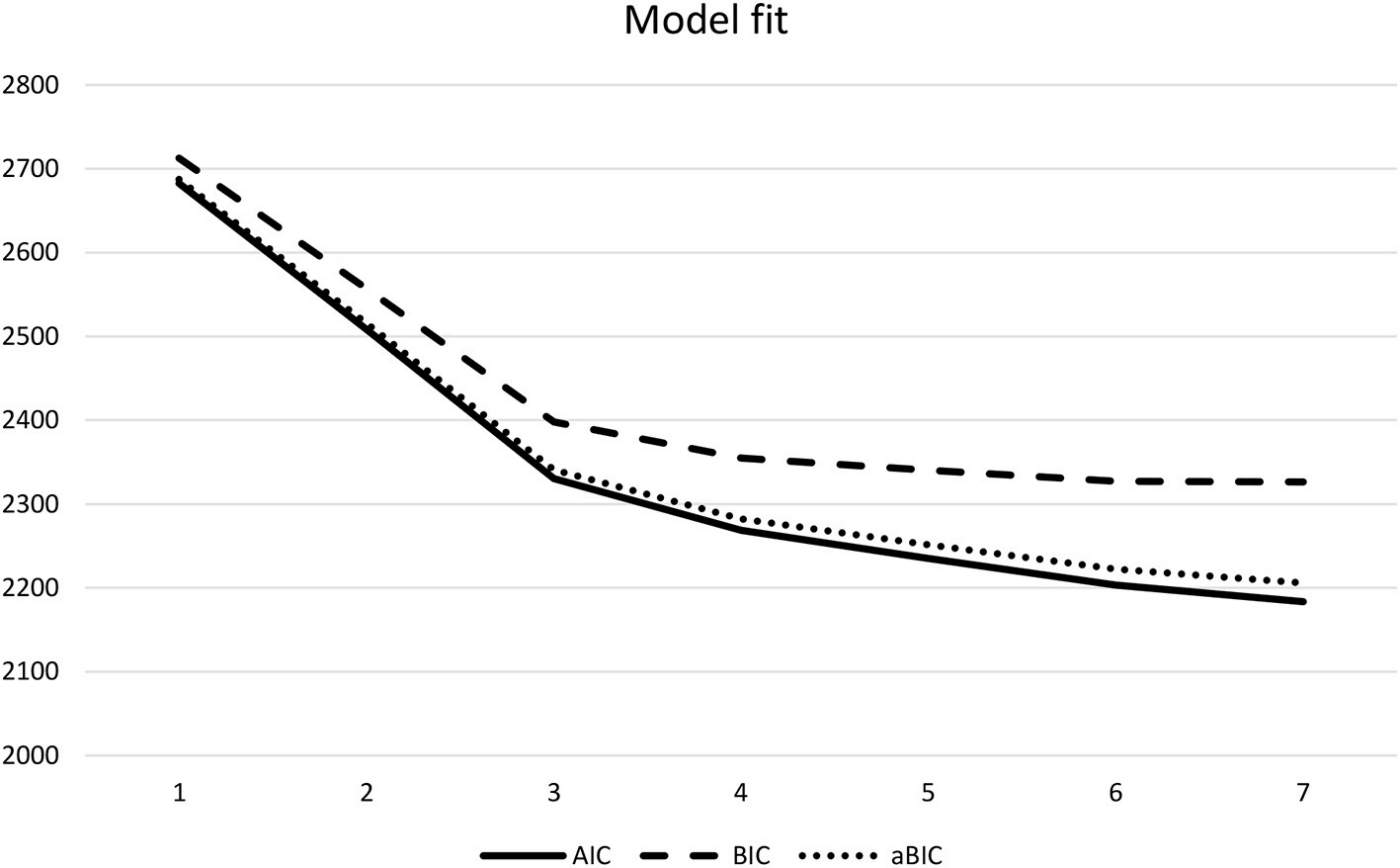
Latent profile analysis: model fit. AIC, Akaike information criterion; BIC, Bayesian information criterion; aBIC, sample-size adjusted BIC.

**Figure 2:**
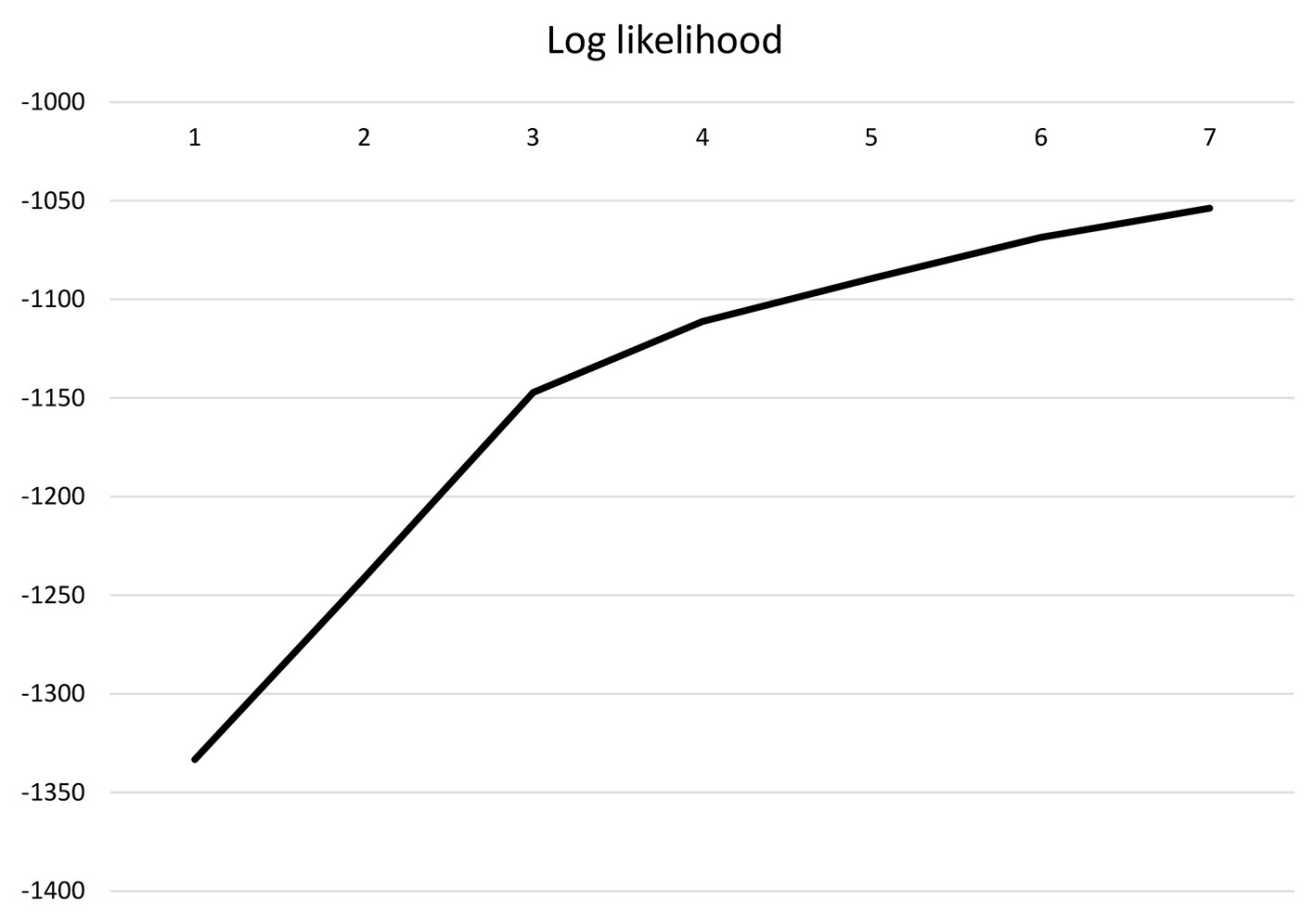
Latent profile analysis: log likelihood.

To answer the second research question, adolescents' mutual acculturation attitudes were analyzed through latent profile analyses (LPA) and the four acculturation dimensions were used as continuous variables. Namely, attitudes toward (1) migration-background adolescents' heritage culture maintenance, (2) migration-background adolescents' dominant culture adoption, (3) majority adolescents' acquisition of cultural knowledge, and (4) schools' endorsement of intercultural contact were analyzed. Model fit indices (see Table 3) and theoretical considerations guided model selection (Geiser, 2009). Whereas, model fit values decreased from the one-profile-solution to the seven-profile-solution, the decrease extenuated after the three-profile-solution (see Figures 1, 2 for the LPA elbow plot on AIC, BIC, aBIC, and log likelihood values). Entropy values were adequate for all profiles, yet best for the four- and three-profile-solutions. Whereas, the Lo-Mendell-Rubin adjusted log-likelihood-ratio test (aLMR LRT) did not indicate a significant model fit improvement between one and two profiles, it indicated a significant model fit improvement between three and two profiles as well as between four and three profiles. However, given the small number of participants in one of the four profiles (3%, *n* = 8) and the rule of deference to more constrained and parsimonious models (Lanza et al., 2013), the three-profile-solution was chosen.

**Table 3:** Overview model fit latent profile analyses.

**No**.	**Log likelihood**	**AIC**	**BIC**	**aBIC**	**Entropy**	**aLMR, *p*-value**	**BLRT, *p*-value**	**Sample proportion per class**	**Classification accuracy**
1	−1333.281	2682.563	2712.634	2687.260				317 (100%)	
2	−1241.226	2508.452	2557.318	2516.085	0.810	0.09	<0.001	49 (16%); 268 (85%)	0.900–0.955
**3**	**−1147.108**	**2330.217**	**2397.877**	**2340.785**	**0.917**	**<0.001**	**<0.001**	**33 (10%); 137 (43%); 147 (46%)**	**0.955–0.964**
4	−1111.247	2268.494	2354.949	2281.998	0.933	0.04	<0.001	8 (3%); 29 (9%); 146 (46%); 134 (42%)	0.954–0.989
5	−1089.431	2234.861	2340.111	2251.301	0.874	0.05	<0.001	29 (9%); 82 (26%); 8 (3%); 137 (43%); 61 (19%)	0.882–0.985
6	−1068.587	2203.174	2327.218	2222.550	0.879	0.32	<0.001	7 (2%); 78 (25%); 132 (42%); 10 (3%); 29 (9%); 61 (19%)	0.849–0.995
7	−1053.752	2183.504	2326.342	2205.815	0.886	0.44	<0.001	10 (3%); 7 (2%); 26 (8%); 78 (25%); 5 (2%); 129 (41%); 62 (20%)	0.848–0.999

AIC, Akaike information criterion; BIC, Bayesian information criterion; aBIC, sample-size adjusted BIC; aLMR LRT, Lo–Mendell–Rubin adjusted log-likelihood-ratio test; BLRT, bootstrap likelihood ratio test. Classification accuracy relates to the average latent class probabilities. The chosen profile solution is in bold.

**Figure 3:**
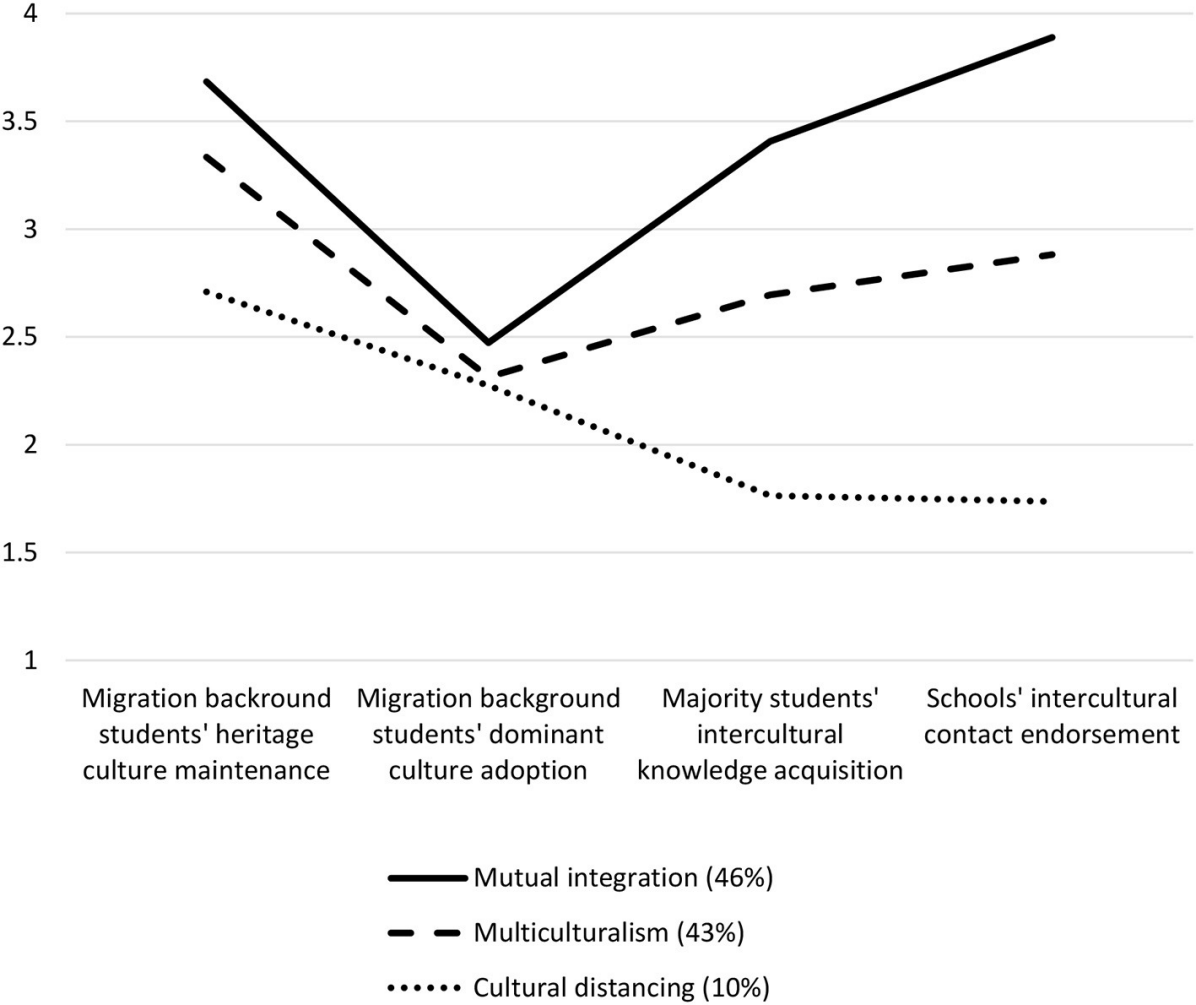
Mutual acculturation profiles assessed via latent profile analysis (*n* = 317). Mutual acculturation profiles were assessed through two minority dimensions (migration background students' heritage culture maintenance and dominant culture adoption) and two majority dimensions (majority students' intercultural knowledge acquisition and schools' intercultural contact endorsement) on a four-point Likert scale with 1 = *disagree*, 2 = *somewhat disagree*, 3 = *somewhat agree*, 4 = *agree*.

Figure 3 displays the three distinct profiles, and Table 4 shows the exact agreement values of participants within each of the three profiles concerning all four acculturation dimensions. The *mutual integration profile* (*n* = 147; 46%) is characterized by a strong agreement with migration-background students' heritage culture maintenance, majority students' acquisition of intercultural knowledge, and schools' endorsement of intercultural contact as well as a tendency to agree with migration-background students' dominant culture adoption. The *multiculturalism profile* (*n* = 137; 43%) is characterized by agreement with migration-background students' heritage culture maintenance, a tendency to agree with the two majority dimensions, and a tendency to disagree with migration-background students' dominant culture adoption. The *cultural distancing* profile (*n* = 33, 10%) could also be called a separation profile because it is characterized by a tendency to agree with migration-background students' heritage culture maintenance, a tendency to disagree with migration-background students' dominant culture adoption, and disagreement with the two majority dimensions.

**Table 4:** Mean values for each acculturation dimension of each acculturation profile.

**Profile**	***n***	**Heritage culture maintenance**		**Dominant culture adoption**		**Intercultural knowledge acquisition**		**Intercultural contact endorsement**	
		**M**	**SE**	**M**	**SE**	**M**	**SE**	**M**	**SE**
Cultural distancing	33 (10%)	2.71	0.185	2.28	0.155	1.76	0.139	1.74	0.089
Multiculturalism	137 (43%)	3.33	0.049	2.31	0.057	2.70	0.054	2.88	0.029
Mutual integration	147 (46%)	3.68	0.035	2.47	0.086	3.41	0.050	3.89	0.016

Answer scale ranges from disagree (1) to agree (4).

### 7.3. Mutual acculturation profiles and national self-identification

To answer the third research question, I assessed group differences concerning the three mutual acculturation profiles using univariate ANOVA and multiple logistic regression. Each profile revealed by LPA was assessed in relation to participants' self-identification as belonging to the Swiss majority. This national belonging was assessed according to self-identification as being Swiss or as self-identification as having a migration background.

Table 5 displays mean values for each self-identification assessment (being Swiss, and not having a migration background) of each acculturation profile. There were no significant differences among the three profiles in terms of self-identification as being Swiss: *F*_(2, 312)_ = 2.27, *p* = 0.105, η^2^ = 0.014. This means that self-identification in terms of being Swiss did not significantly differ across the three mutual acculturation profiles. There were significant mean differences in terms of self-identification as having a migration background, *F*_*Welch*_(2, 89.99) = 4.70, *p* = 0.006, η^2^ = 0.031. Based on a Games-Howell *post-hoc* test, those in the cultural distancing profile were found to self-identify significantly stronger as not having a migration background (*M* = 3.45, *SD* = 1.03) than those in the mutual integration profile (*M* = 2.74, *SD* = 1.27, *p* = 0.005). No further significant differences arose across the mutual acculturation profiles in terms of migration background self-identification. I ran sensitivity analyses in G^*^Power (Version 3.1.19.7) and found that an ANOVA with 297–315 participants across 3 groups without covariates is sensitive to effects of 0.22–0.23 magnitude with 80% power (alpha = 0.05). Thus, effects with an effect size smaller than 0.22 could not be reliably detected in this study.

**Table 5:** Mean values for each self-identification assessment of each acculturation profile.

**Profile**	**Being Swiss**		**Not having a migration background**	
	**M (SD)**	***n***	**M (SD)**	***n***
Cultural distancing	3.33 (1.02)	33	3.45 (1.03)	31
Multiculturalism	2.89 (1.14)	137	2.98 (1.16)	130
Mutual integration	2.96 (1.03)	145	2.74 (1.27)	136

Self-identification as Swiss was rated on a scale from 1 = not being Swiss to 4 = being Swiss. Self-identification as having a migration background was rated on a scale from 1 = having a migration background to 4 = not having a migration background.

Table 6 displays three multiple logistic regression analyses. A significant effect was found between the cultural distancing and the mutual integration profiles in terms of self-identification as not having a migration background, *b* = 0.519, *SE* = 0.230, *OR* = 1.681, *p* = 0.024, 95% [1.07, 2.64]. Thus, the log odds of self-identifying as not having a migration background were significantly higher in the cultural distancing profile than in the mutual integration profile, confirming the findings of the ANOVA. However, the results indicated no significant interaction between self-identification as being Swiss and as not having a migration background in relation to mutual acculturation profile membership (see Annex Figures A7–A9 for interaction effect graphs). Simple effect coefficients were computed on three values of Swiss self-identification, first for the mean-centered variable, then 1 SD above the mean, and 1 SD below the mean. When self-identification as being Swiss was fixed 1 SD above and below the mean, the difference between the cultural distancing and the mutual integration profiles in terms of self-identification as not having a migration background was not significant anymore. All in all, these results indicate that those in the cultural distancing profile self-identified stronger as not having a migration background than those in the mutual integration profile, but self-identification as being Swiss and the interaction between being Swiss and not having a migration background were not a distinctive feature of the three mutual acculturation profiles.

**Table 6:** Multiple logistic regressions.

	**Model**	**Swiss centered**				**Swiss low**				**Swiss high**			
**Profile**		**B**	**SE**	**Wald**	**OR [C.I. 95%]**	**B**	**SE**	**Wald**	**OR [C.I. 95%]**	**B**	**SE**	**Wald**	**OR [C.I. 95%]**
Multi-culturalism (*n* = 130) vs. mutual integration (*n* = 136)	Intercept	0.004	0.134	0.001		−0.143	0.187	0.587		0.151	0.197	0.586	
	No mbg	0.189	0.112	2.855	1.209 [0.97, 1.51]	0.106	0.164	0.420	1.112 [0.81, 1.53]	0.272	0.143	3.615	1.313 [0.99, 1.74]
	Swiss	−0.136	0.127	1.137	0.873 [0.68, 1.12]	−0.136	0.127	1.137	0.873 [0.68, 1.12]	−0.136	0.127	1.137	0.873 [0.68, 1.12]
	No mbg ^*^ Swiss	−0.077	0.098	0.618	0.926 [0.77, 1.12]	−0.077	0.098	0.618	0.926 [0.77, 1.12]	−0.077	0.098	0.618	0.926 [0.77, 1.12]
Cultural distancing (*n* = 31) vs. Mutual integration (*n* = 136)	Intercept	−1.650^***^	0.253	42.551		−1.512^***^	0.359	17.728		−1.788^***^	0.373	22.927	
	No mbg	0.519^*^	0.230	5.100	1.681 [1.07, 2.64]	0.587	0.338	3.027	1.799 [0.93, 3.49]	0.451	0.296	2.314	1.569 [0.88, 2.81]
	Swiss	0.128	0.245	0.272	1.136 [0.70, 1.84]	0.128	0.245	0.272	1.136 [0.70,0.1.84]	0.128	0.245	0.272	1.136 [0.70,0.1.84]
	No mbg ^*^ Swiss	0.063	0.202	0.097	1.065 [0.72, 1.58]	0.063	0.202	0.097	1.065 [0.72, 1.58]	0.063	0.202	0.097	1.065 [0.72, 1.58]
Multi-culturalism (*n* = 130) vs. cultural distancing (*n* = 31)	Intercept	1.654^***^	0.252	43.012		1.369^***^	0.363	14.241		1.939^***^	0.366	28.126	
	No mbg	−0.330	0.231	2.042	0.719 [0.46, 1.13]	−0.481	0.342	1.985	0.618 [0.32, 1.21]	−0.178	0.294	0.368	0.837 [0.47, 1.49]
	Swiss	−0.263	0.243	1.178	0.769 [0.48, 1.24]	−0.263	0.243	1.178	0.769 [0.48, 1.24]	−0.263	0.243	1.178	0.769 [0.48, 1.24]
	No mbg ^*^ Swiss	−0.140	0.203	0.475	0.870 [0.58, 1.29]	−0.140	0.203	0.475	0.870 [0.58, 1.29]	−0.140	0.203	0.475	0.870 [0.58, 1.29]
	Cox and Snell	0.043				0.043				0.043			
	Nagelkerke	0.051				0.051				0.051			

No mbg, no migration background self-identification; Swiss, Swiss self-identification. ^*^p ≤ 0.05. ^***^p ≤ 0.001.

## 8. Discussion

This study was aimed at assessing whether adolescents differ in their mutual acculturation attitudes based on their national self-identification as being Swiss and/or having a migration background. Whereas, mutual acculturation attitudes have already been assessed in the school context (Sidler et al., 2022), how adolescents' self-identification relates to these mutual acculturation attitudes presented a research gap. Self-identification measures are key because in defining individuals' categorization in relation to ongoing norms of national belonging, researchers implicitly play a role in defining belonging and otherness (Moffitt and Juang, 2019). Descriptive results in this study showed that even though most students who were born in Switzerland or who have the Swiss nationality self-identified as being Swiss, there are adolescents who despite having been born in Switzerland or having the Swiss nationality do not self-identify as being Swiss. Moreover, concerning the combination of self-identifying as being Swiss and having a migration background, 31% stated clearly that they identify as being Swiss while not having a migration background. However, various combinations of being Swiss and having a migration background were found for the remaining students, showing that there are diverse degrees of national self-identification. Addressing self-identification when categorizing study participants matters particularly in acculturation research, because acculturation entails a negotiation of dominance (Zick, 2010). Where the chosen constructs and narratives can foster otherness (Tekin, 2010), they can also be used to enhance mutuality. Therefore, it is vital to be aware of the potential impact of group-based terminology. Thus, assessing mutual acculturation attitudes in terms of national self-identification promised novel insights through giving the authority of interpretation concerning minority and majority group members categorization to the study participants themselves.

Latent profile analyses led to three mutual acculturation profiles: The *mutual integration profile* was characterized by strong agreement concerning migration-background students' heritage culture maintenance, schools' endorsement of intercultural contact, and Swiss adolescents' intercultural knowledge acquisition. Thus, just as acculturation is a mutual process between minority and majority group members, integration goes beyond expecting only openness from majority group members as it is stated in the Federal Act on Foreign Nationals, SR 142.20 (2005). Adolescents in the mutual integration profile were indecisive concerning the adoption dimension (namely, migration-background adolescents' dominant culture adoption), meaning that they agreed with the adoption of some issues and disagreed with the adoption of others. Because the final adoption score reflects the mid-point of the 4-point Likert answer scale, this pattern is still considered to be an integration pattern. The *multiculturalism* profile was defined by agreement with the heritage culture maintenance dimensions; however, this profile featured a tendency to agree with only the two majority dimensions and a tendency to disagree with the dominant culture adoption dimension. Multiculturalism refers to a society in which various distinct cultural groups are considered to be relevant and given the agreement concerning migration background students' heritage culture maintenance and the tendency to agree that majority students' intercultural knowledge acquisition and schools' intercultural contact endorsement are important, this pattern was defined as a multiculturalism pattern. Finally, the *cultural distancing* profile demonstrated a tendency to agree with the heritage culture maintenance dimension and a tendency to disagree with the dominant culture adoption dimension and disagreement with both majority dimensions. The three profiles found in this study resemble the three profiles found in the first year of the longitudinal data collection (Sidler et al., 2022), showing only a slight variation in the *cultural distancing* profile in terms of shifting from disagreement to agreement concerning migration background students' heritage culture maintenance.

The three mutual acculturation profiles present four striking insights concerning adolescents' mutual acculturation attitudes: First, students across the three profiles agreed to a different degree with migration-background adolescents' heritage culture maintenance. This means that there is a general acceptance and tolerance of heritage cultures and therefore of cultural diversity among adolescents in schools in the German-speaking cantons Aargau, Basel-Stadt, and Solothurn in Switzerland. This development toward agreement with migration background students' heritage culture maintenance could be explained by participants' age because adolescents' openness to diverse views has been found to be gradually increasing in another European context (Bayram Özdemir et al., 2021). Interestingly, adolescents in all three profiles not only agree or tend to agree with migration-background adolescents' heritage culture maintenance but also show indifference regarding or a tendency to disagree with migration-background students' dominant culture adoption. The combination of finding migration background students' heritage culture maintenance important while being indifferent toward their dominant culture adoption could also relate to tolerance instead of exclusionary practices found in other studies (Duemmler, 2015). Then, just as the year before, most adolescents (46%) in this study were in the mutual integration profile, followed by 43% in the multiculturalism profile. The strong prevalence of the mutual integration profile and majority acculturation as a distinctive feature of the three profiles stress the importance of a mutual acculturation framework at school in the Swiss context. Finally, it is interesting to note that the three profiles vary most concerning the two majority acculturation dimensions, indicating that majority acculturation is a distinctive feature of the three patterns. Thus, the patterns clearly show the added value of a mutual acculturation framework, combining minority and majority acculturation when assessing acculturation attitudes.

One of the characteristics of the mutual integration and the multiculturalism profiles is the importance students place on schools endorsing intercultural contact and exchange. This is an important finding because schools supporting positive intercultural contact and cultural diversity discussions promote intercultural understanding, which in turn prepares students to participate in a culturally diverse society (Schachner et al., 2021). Moreover, promoting discussions and intercultural contact may also support students in forming intergroup friendships (Schachner et al., 2015), which in turn may again enhance intercultural understanding. Additionally, adolescents' development is influenced by schools' organizational contexts and cultural diversity climates (Juang and Schachner, 2020), particularly in terms of students' acculturation and school adjustment (Schachner et al., 2017). Thus, in addition to providing an important acculturation context (Arends-Tóth and Van de Vijver, 2006), schools serve as important acculturation agents in adjusting their cultural diversity policies to the needs of adolescents with and without migration backgrounds to promote their intercultural competencies and development (Sidler et al., 2022). Practically, these findings stress the importance for adolescents that schools implement diversity policies that include endorsing intercultural contact and exchange for students with and without a migration background. Thus, schools should promote intercultural understanding through creating space for students with and without a migration background to discuss cultural diversity and foster exchange about local and heritage cultures.

The three identified mutual acculturation profiles were then analyzed in a second step through an ANOVA and multiple logistic regressions in terms of national self-identification. To avoid categorizing adolescents using a top-down approach through categorizations such as migration background based on nationality and countries of birth of adolescents and their parents, the adolescents were asked to self-identify as belonging to the majority in terms of either being Swiss or having a migration background. Those in the cultural distancing profile were found to identify significantly stronger as not having a migration background than those in the mutual integration profile. No significant interaction effect concerning self-identification as being Swiss and as not having a migration background emerged. Next to being significant effects, the effect size, and the amount of explained variance were not minuscule. Two main findings are to be discussed:

First, adolescents in the cultural distancing profile identified significantly stronger as not having a migration background and therefore as belonging to the dominant majority than those in the mutual integration profile. The cultural distancing profile differs from the mutual integration profile in two important aspects: a separation expectation toward minority group members (instead of an integration expectation) and a non-involvement expectation toward majority group members and schools (instead of an integration expectation). Thus, there were exclusionary and distancing tendencies in the cultural distancing profile in that minority group adolescents were not expected to adopt dominant cultural characteristics in Switzerland while Swiss adolescents and Swiss schools were not expected to acquire intercultural knowledge and foster intercultural contact. Adolescents expecting minority group members to separate were more likely to self-identify as belonging to the national majority compared to adolescents expecting minority group members to integrate. This aligns with previous findings in Switzerland concerning students reproducing exclusionary and/or assimilationist practices (Duemmler, 2015). Then, adolescents who had low expectations of the majority to integrate in terms of majority students acquiring intercultural knowledge and schools endorsing intercultural contact were more likely to self-identify as not having a migration background compared to adolescents expecting majority group members and schools to integrate. Thus, having low expectations on the majority to integrate was related to whether one self-identified as belonging to this majority. This finding relates to Swiss integration policies in that although the Swiss majority is expected to be open-minded, members of the Swiss majority are not expected to actively integrate (Federal Act on Foreign Nationals, SR 142.20, 2005). Just as acculturation research has focused predominantly on minority acculturation, national integration policies have focused on how minorities and migrants should integrate in the dominant society. The role majority group members and institutions play concerning the social inclusion of all residents, whether they are majority or minority group members, is therefore largely ignored. Whereas, the link between the cultural distancing profile and self-identifying as not having a migration background was significant, future studies are needed to define the directionality of the association.

The second finding is that there was no significant difference in terms of national self-identification between adolescents in the multiculturalism profile and those in the mutual integration profile. Moreover, there was also no significant difference between all three mutual acculturation profiles concerning the interaction of the two self-identification measures. Thus, national self-identification measures were not a distinctive characteristic concerning mutual acculturation attitudes *per se*. This could be explained by the age of the participants, as they develop their own labels and try out various identities during adolescence (Portes and Rivas, 2011). Additionally, adolescents might have experienced cultural diversity at earlier ages and therefore developed more open-minded attitudes. However, the sample size could have been too small to find more meaningful effects. Moreover, it could also be explained by the one-sided assessment of self-identification because this study's focus was to measure national self-identification and not self-identification in its diversity. The diversity of experiences among immigrants and their descendants has been well-documented (Moffitt and Juang, 2019), and assessing more diverse and more nuanced self-identifications particularly in relation to diverse minority self-identifications might have led to further insights. The same might apply for majority group members because they are affected by not only mutual but also remote acculturation (Ferguson and Bornstein, 2012). Whereas, self-identification measures surely help de-essentialize the national/majority and the minority groups, they may rely heavily on internalized social power structures, as these findings could suggest. Self-identification measures therefore do not magically overcome issues of societal power structures and dominance, as adolescents may just as well refer to the same societal power structures when they define themselves—particularly if they were socialized in the relevant society—and base their national self-identification on how they are perceived in their environment.

## 9. Limitations

There are five main limitations to this study: first, the sample size has been a limiting factor. LPAs for subsamples based on self-identification as being Swiss or as having a migration background were not meaningful for both relevant subsamples due to the small sample sizes for those self-identifying as not being Swiss and for those self-identifying as having a migration background. Thus, to assess mutual acculturation patterns based on self-identification and therefore to assess latent mutual acculturation patterns directly for the diverse groups, future research with a bigger sample is needed. Additionally, the sample size was also a limiting factor in accounting for diverse migration backgrounds and thus diverse ethnic self-identifications. Therefore, this study is limited by the specific national self-identification assessment, namely self-identification as (1) being Swiss, (2) having a migration background, and (3) the interaction of the two. Whereas, this study's focus lied on self-identification within national power structures, further research could assess national and ethnic self-identification and their combinations in a more nuanced way. Specifically, diverse ethnic self-identifications (e.g., specific countries or regions), binational identifications (e.g., one parent with the Swiss nationality, the other not; both parents with different nationalities outside of Switzerland; or parents with multiple nationalities), as well as internal migration and therefore cantonal self-identifications (Switzerland has four official languages and a strong federal system with cantonal regions) could be studied. Similarly, the mutual acculturation measurement assesses attitudes toward minority and majority groups to focus on social power structures. However, it could be further developed to measure mutual acculturation attitudes toward specific minority groups (like specific nationalities, specific cantons or linguistic regions, binational adolescents, …). This would allow to assess attitudes of specific minority groups toward other minority groups as well as attitudes of the majority group toward different minority groups. Stereotyping and discriminating attitudes can only be discovered through such specified data. Then, as adolescents' self-identifications develop over time, a longitudinal assessment would yield better understanding. Finally, further research is needed to assess self-identification within societal power structures and understand the impact of dominant societal narratives on not only adolescents' self-identification and development thereof but also concerning how self-identification might shape mutual acculturation attitudes or vice versa.

## 10. Conclusion

This study's main aim was to assess whether adolescents' mutual acculturation attitudes varied depending on their national self-identification. Mutual acculturation is a process by which members of minority and majority groups—the latter including national institutions—adjust to and change because of intercultural encounters. Minority and majority perspectives are frequently used to examine acculturation attitudes; however, researchers frequently distinguish between minority and majority group members based on concepts such as place of birth, nationality, migrant generation, or migration background. However, researchers may classify members of a group differently than the group's members self-identify. Adolescents' *mutual acculturation attitudes* have so far not been studied in relation to national self-identification measures. This study addressed this research gap by examining adolescents' mutual acculturation attitudes in relation to whether they self-identified as being Swiss, as having a migration background, and the interaction of the two. With latent profile analyses, I identified three mutual acculturation profiles: (1) a mutual integration profile—where migration-background and Swiss adolescents and schools are expected to integrate—and (2) a multiculturalism profile—considering diverse cultural groups as well as intercultural knowledge and contact as important—as well as (3) a cultural distancing profile with separation expectations toward minority adolescents and non-involvement expectations toward majority adolescents and schools. Across the three profiles I found a general acceptance of cultural diversity. Moreover, the three profiles vary most concerning the two majority acculturation dimensions, indicating that the acculturation of majority group members and schools is a distinctive feature of the three patterns. Thus, the patterns clearly show the added value of a mutual acculturation framework, which combines minority, majority, and institutional acculturation. Concerning mutual acculturation attitudes and national self-identification, I found that those in the cultural distancing profile self-identified significantly stronger as not having a migration background than those in the mutual integration profile. However, as this was a cross-sectional study, the direction of this link is unclear and could be assessed by future studies.

## Data availability statement

The raw data supporting the conclusions of this article will be made available by the authors, without undue reservation.

## Ethics statement

The studies involving human participants were reviewed and approved by Ethics Committee of the Faculty of Arts and Social Sciences of the University of Zurich. Written informed consent to participate in this study was provided by the participants' legal guardian/next of kin.

## Author contributions

PS participated in the study design and data collection, performed the statistical analysis and data interpretation, and wrote the manuscript.

## Footnotes

^1^Switzerland's nationality laws follow the jus sanguinis principle by which citizenship is determined by the nationality of one or both parents. Moreover, as stated in the chapter on acculturation context, the MIPEX found Switzerland to have high naturalization barriers. This means that many non-Swiss citizens have lived for a long time or have even been born in Switzerland and shows the importance of self-identification measures in the Swiss context.
