# Supplementary material for: Adolescents' mutual acculturation attitudes and their association with national self-identification in three Swiss cantons
Source: Front Sociol. 2023 Jun 21;8:953914. doi: 10.3389/fsoc.2023.953914 (PMC10320855; doi:10.3389/fsoc.2023.953914)
Supplement: Supplementary file 1 [file Table_1.docx]

# 19 Annex

**Table 1A***Assessment of Mutual Acculturation Attitudes: Items Verbatim*

| **Dimension** | **Migration background students’ heritage culture maintenance** | **Migration background students’ dominant culture adoption** | **Majority students’ acquisition of cultural knowledge** | **Schools’ endorsement of intercultural contact** |
| --- | --- | --- | --- | --- |
| Introduction | I find that it is important for teenagers from another country who live in Switzerland … | | I find it is important that Swiss teenagers who live in Switzerland have to get to know… | I find it is important that the Swiss schooling system allows teenagers from other countries and Swiss teenagers… |
| Item 1 | …to be allowed to preserve their traditions and customs. | …to adopt the dominant traditions and customs in Switzerland. | … the traditions and customs of teenagers from other countries who live in Switzerland. | … to exchange information about their traditions and customs. |
| Item 2 | …to be allowed to preserve their religion. | …to adopt the dominant religion in Switzerland. | … the religions of teenagers from other countries who live in Switzerland. | … to exchange information about their religions. |
| Item 3 | …to be allowed to preserve their way of life. | …to adopt the dominant way of life in Switzerland. | … the ways of life of teenagers from other countries who live in Switzerland. | … to exchange information about their ways of life. |
| Item 4 | …to be allowed to preserve their language. | …to adopt (one of) the official language in Switzerland. | … the languages of teenagers from other countries who live in Switzerland. | … to exchange information about their languages. |
| Item 5 | …to be allowed to preserve their way of clothing. | …to adopt the dominant way of clothing in Switzerland. | … the ways of clothing of teenagers from other countries who live in Switzerland. | … to exchange information about their ways of clothing. |
| Item 6 | …to be allowed to preserve their family culture. | …to adopt the dominant family culture in Switzerland. | … the family cultures of teenagers from other countries who live in Switzerland. | … to exchange information about their family cultures. |
| Item 7 | …to be allowed to preserve their views on professional careers of women and men. | …to adopt the dominant views on professional careers of women and men in Switzerland. | … the views on professional careers of women and men of teenagers from other countries who live in Switzerland. | … to exchange information about their views on professional careers of women and men. |

**Table 2A**

*Overview Model Fit Latent Profile Analyses for two subsamples: those self-identifying as (somewhat) being Swiss (n = 213) and those who self-identified as (somewhat) not being Swiss (n = 102)*

| Swiss | # | Log likelihood | AIC | BIC | aBIC | Entropy | aLMR  *p*-value | BLRT  *p*-value | Sample proportion per class | Classification accuracy |
| --- | --- | --- | --- | --- | --- | --- | --- | --- | --- | --- |
| Yes | 1 | -918.802 | 1853.604 | 1880.494 | 1855.144 |  |  |  | 213 (100%) |  |
|  | 2 | -857.006 | 1740.012 | 1783.709 | 1742.515 | .680 | .40 | < .001 | 139 (65%); 74 (35%) | .850–.927 |
|  | 3 | -789.648 | 1615.297 | 1675.800 | 1618.763 | .913 | < .01 | < .001 | 24 (11%); 91 (43%); 98 (46%) | .956–.978 |
|  | 4 | -761.333 | 1568.667 | 1645.976 | 1573.096 | .930 | .10 | < .001 | 7 (3%); 97 (46%); 21 (10%); 88 (42%) | .958–.984 |
|  | 5 | -743.177 | 1542.355 | 1636.471 | 1547.747 | .877 | .18 | < .001 | 7 (3%); 21 (10%); 44 (21%); 51 (24%); 90 (42%) | .872–.979 |
|  | 6 | -726.503 | 1519.005 | 1629.928 | 1525.361 | .890 | .13 | < .001 | 5 (2%); 19 (10%); 90 (42%); 6 (3%); 43 (20%); 50 (24%) | .882–.999 |
| No | 1 | -398.862 | 813.723 | 834.723 | 809.454 |  |  |  | 102 (100%) |  |
|  | 2 | -363.072 | 752.143 | 786.268 | 745.206 | .881 | .58 | < .001 | 79 (78%); 23 (23%) | .918–.979 |
|  | 3 | -337.876 | 711.753 | 759.002 | 702.147 | .959 | .12 | < .001 | 24 (24%); 3 (3%); 75 (74%) | .975–.993 |
|  | 4 | -326.116 | 698.232 | 758.607 | 685.958 | .868 | .36 | < .001 | 3 (3%); 24 (24%); 55 (54%); 20 (20%) | .890–.998 |
|  | 5 | -315.735 | 687.470 | 760.969 | 672.528 | .910 | .58 | < .001 | 20 (20%); 4 (4%); 52 (51%); 23 (23%); 3 (3%) | .889–.995 |
|  | 6 | -302.112 | 670.223 | 756.847 | 652.612 | .936 | .56 | < .001 | 2 (2%); 25 (25%); 4 (4%); 8 (8%); 41 (40%);22 (22%) | .913–1.00 |

*Note.* Lo–Mendell–Rubin adjusted log-likelihood-ratio test; BLRT = bootstrap likelihood ratio test. Classification accuracy relates to the average latent class probabilities.

**Table 3A**

*Overview Model Fit Latent Profile Analyses for two subsamples: those self-identifying as (somewhat) having a migration background (n = 102) and those who self-identified as (somewhat) not having a migration background (n = 195)*

| Migration background | # | Log likelihood | AIC | BIC | aBIC | Entropy | aLMR  *p*-value | BLRT  *p*-value | Sample proportion per class | Classification accuracy |
| --- | --- | --- | --- | --- | --- | --- | --- | --- | --- | --- |
| Yes | 1 | -405.701 | 827.403 | 848.403 | 823.134 |  |  |  | 102 (100%) |  |
|  | 2 | -377.262 | 780.524 | 814.649 | 773.586 | .870 | .27 | < .001 | 45 (43%); 57 (57%) | .953–.963 |
|  | 3 | -338.068 | 712.136 | 759.386 | 702.530 | .952 | .12 | < .001 | 6 (7%); 41 (39%); 55 (54%) | .956–1.00 |
|  | 4 | -325.238 | 696.477 | 756.851 | 684.203 | .902 | .40 | < .001 | 42 (39%); 6 (7%); 30 (29%); 24 (25%) | .919–1.00 |
|  | 5 | -310.564 | 677.129 | 750.628 | 662.186 | .925 | .19 | < .001 | 42 (40%); 30 (29%); 2 (2%); 4 (5%); 24 (25%) | .925–1.00 |
|  | 6 | -296.917 | 659.834 | 746.459 | 642.224 | .938 | .52 | < .001 | 28 (27%); 8 (8%); 2 (2%); 5 (6%); 39 (37%); 20 (21%) | .927–1.00 |
| No | 1 | -838.882 | 1693.764 | 1719.948 | 1694.605 |  |  |  | 195 (100%) |  |
|  | 2 | -781.262 | 1588.523 | 1631.072 | 1589.890 | .794 | .07 | < .001 | 36 (21%); 159 (79%) | .933–.950 |
|  | 3 | -727.791 | 1491.583 | 1550.497 | 1493.475 | .902 | < .01 | < .001 | 79 (40%); 24 (13%); 92 (47%) | .951–.956 |
|  | 4 | -701.965 | 1449.931 | 1525.210 | 1452.349 | .923 | .13 | < .001 | 24 (13%); 6 (3%); 78 (40%); 87 (44%) | .947–.996 |
|  | 5 | -687.053 | 1430.107 | 1521.751 | 1433.051 | .931 | .33 | < .001 | 5 (3%); 84 (42%); 76 (40%); 24 (13%); 6 (4%) | .930–.995 |
|  | 6 | -673.211 | 1412.422 | 1520.431 | 1415.892 | .934 | .64 | < .001 | 5 (3%); 7 (3%); 77 (39%); 23 (12%); 3 (2%); 80 (40%) | .861–.999 |

*Note.* Lo–Mendell–Rubin adjusted log-likelihood-ratio test; BLRT = bootstrap likelihood ratio test. Classification accuracy relates to the average latent class probabilities.
