# Supplementary material for: Adolescents' mutual acculturation attitudes and their association with national self-identification in three Swiss cantons
Source: Front Sociol. 2023 Jun 21;8:953914. doi: 10.3389/fsoc.2023.953914 (PMC10320855; doi:10.3389/fsoc.2023.953914)
Supplement: Supplementary file 2 [file Data_Sheet_1.docx]

# 19 Annex

**Figure 1A**

*Swiss Self-Identification and Student’ Nationality*

**Figure 2A**

*Swiss Self-Identification and Student’ Country of Birth*

**Figure 3A**

*Swiss Self-Identification and Parents’ Country of Birth*

**Figure 4A**

*Migration Background Self-Identification and Student’ Nationality*

**Figure 5A**

*Migration Background Self-Identification and Student’ Country of Birth*

**Figure 6A**

*Migration Background Self-Identification and Parents’ Country of Birth*

**Figure 7A**

*Interaction Self-identification Being Swiss * Not Having Migration Background
Multiculturalism vs. Mutual Integration*

**Figure 8A**

*Interaction Self-identification Being Swiss * Not Having Migration Background
Cultural Distance vs. Mutual Integration*

**Figure 9A**

*Interaction Self-identification Being Swiss * Not Having Migration Background
Multiculturalism vs. Cultural Distance*
